# **Keyword index to Volume 83**

**DOI:** 10.1054/bjoc.2000.1592

**Published:** 2000-12


					British Journal of Cancer (2000) 83(12), 1778-1781 (c) 2000 Cancer Research Campaign
Accessory cells 261
Acquired resistance 270
Activation 1387
Activation systems 1351
Active surveillance 1249
Activity 998
Acute lymphoblastic leukaemia
756, 1617
Acute myeloid leukaemia 91
Adenocarcinoma 632
S-Adenosylmethionine
decarboxylase 594
Adjusted analysis 1338
Adjuvant chemotherapy 577
Adjuvant therapy 16
Adolescents 397
Aerosol delivery 935
Aetiology 127
Ageing 841
Alcohol 949
Alcohol consumption 689
Alcoholism 949
Alkylating agents 267, 1291
5-Aminolaevulinic acid 539,
655
2-(4-Aminophenyl)-
benzothiazole 270
Amplification 1020, 1688
Anal canal cancer 1637
Anaplastic carcinomas 1494
Anchorage independence 156
Androgens 74
Angiogenesis 63, 196, 480, 826,
880, 1077, 1154
Angiogenic factors 833
Angiopoietin 1154
Angiotensin II 225
Annexin 83
Anoxia 662
Antagonist G 941
Anthracyclines 438
Antibodies, human 252
Antibody-directed enzyme
prodrug therapy 267
Anticancer therapeutics 1401
Antigen processing 1192
Anti-GnRH antibodies 443
Anti-idiotypic vaccines 1202
Anti-inflammatory agents 112
Antimitotic 83
Anus neoplasia 89
Anxiety 1261
AP-1 782, 941
APC 153
Apoptosis 188, 239, 506,
642, 668, 880, 928, 941,
1039, 1096, 1110, 1223,
1318, 1394, 1715, 1735,
1762
AQ4N 1589
[Arg6
,D-Trp7,9
,Nme
Phe8
]-
Substance P (6-11) 941
Arginine 800
Aromatase 333
Arylamines 412
Asbestos 104
Astrocytomas 407
Ataxia-telangiectasia 1650
Atypical adenomatous
hyperplasia 632
Audits 421
Autoreactive T-cells 1405
BA70 874
Barrett's oesophagus 539
Bax 761, 1735
BB94 1536
Bcl-2 506, 761, 1318
Bcl-2/Bax ratio 1380
bcl-w 668
Best supportive care 447
Binding proteins 1344
Biochemotherapy 980
Biodistribution 232
Biological prognostic factors 847
Bioreduction 1589
Birth order 1234
Birthweight 964
Bispecific antibodies 261
Bladder carcinoma 209, 412
Blood 1664
Bombesin/gastrin-releasing
peptide antagonists 906
Bone lesions 360, 973
Bone marrow 1375
Bracken 914
Brain 1110
Brain metastasis 1281
BRCA1 463
BRCA1 153, 384, 737, 1301,
1318
BRCA1 prevalence 719
BRCA2 463
BRCA2 153, 737, 1301, 1318
Breast cancer 91, 98, 153, 164,
311, 319, 438, 487, 737, 782,
870, 874, 949, 964, 1154,
1231, 1301, 1318, 1405,
1412, 1472, 1479, 1554, 1688
Breast cancer cells 1183
Breast cancer prognosis 1664
Breast cancer risk 1650
Breast neoplasia 112, 1650,
1715
Breast-feeding 127, 1557
Buthionine sulfoximine 267
Bystander effect 1223
c-Abl tyrosine kinases 1360
Caffeine 346
cAMP 333
Cancer cachexia 56, 526, 1055
Cancer registries 952, 1554
Cancer screening 632, 1607
Cancer testis antigens 204
Cancer therapy 906
Capecitabine 22, 583
Carboplatin 858
Carcinoembryonic antigens 1323
Carcinogenics 914
Carcinoids, intestinal 952
Carcinoma in situ 729, 1472
Cardia 391
Cardiac tamponade 858
Case-control studies 104, 112,
689, 1550
Caspases 642
Catalytic topoisomerase
inhibitors 1740
Catecholamines 1630, 1747
-Catenin 1168
Caucasians 1020
CD4+
1055
CD8+
1055
CD26/DPP-IV 1139
CD30 252
CD95 1715
CDHP 141
cDNA expression array 246
Cell adhesion 209
Cell cycles 817, 1039, 1069,
1360
Cell cycle blocks 1762
Cell cycle checkpoints 346
Cell lines 756
Cell morphology 1102
Cell motility 1147
Cell senescence 841
Cell signalling 1223
Cell survival 1102
Cell-matrix interactions 1387
Cellular proliferation 532
c-erbA-2 (TR-2) 1674
c-erbB-2 473
Cervical adenocarcinomas 307
Cervical carcinomas 354, 566,
620, 1659
Cervical neoplasia 1563
Cervical screening 561
c-fos 906
Chain shuffling 252
Chemical chaperone 1735
Chemoprevention 935
Chemo-radiotherapy 1436
Chemosensitivity 941
Chemotherapy 8, 458, 602, 715,
853, 1418, 1594
Childhood 397, 956, 1617
Childhood cancer 602, 1571
Childhood growth 964
Childhood leukaemia 692, 1557
Chromosome 1q 42.2-43 1654
Chromosome 9p21 1707
Chromosome 11p15.5 626
Chromosome 11q 750
Chromosome 17 1295
Chromosome instability 1136
Chromosome translocations
1309
CIN3 1563
9-cis-Retinoic acid 239
13-cis-Retinoic acid 583, 1124
Cisplatin 8, 16, 1128, 1209,
1594, 1612
Cisplatin resistance 1047
c-jun 906
CK-20 1323
Clinical trials 577, 956
CLIP 1405
Clonogenic survival 1375
c-met 668
c-myc 1688
Coagulation cascade 164
Cohort studies 89, 964, 1338
Collagen 650
Colonic cancer 550, 725, 833
Colorectal adenocarcinoma 769
Colorectal cancer 188, 215, 324,
431, 668, 750, 887, 1015,
1139, 1323, 1344, 1643, 1646
Combined treatments 1436
Combretastatin A-4 phosphates
811
Comet assays 1523, 1740
Comparative genomic
hybridization 467, 1295,
1309, 1659
Connective tissue growth factor
756
Continuous infusion 458
Continuous variables 1617
COX-2 324
C-reactive proteins 1442
Crohn's disease 89
CT-2584 HMS 1599
Cutpoint 1617
Cyclin A 30
Cyclosporine 1405
Cyfra 21-1 1696
Cytogenetics 1020
Cytokine subcutaneous
metastasis 1453
Cytokines 637, 1367
Cytotoxicity 817, 1069
Day-care attendances 1557
Death 800
del(1p) 40
Dendritic cells 506
Dentition 1238
Keyword index to Volume 83
British Journal of Cancer (2000) 83(12), 1778-1781
(c) 2000 Cancer Research Campaign
doi: 10.1054/ bjoc.2000.1592, available online at http://www.idealibrary.com on
Deoxyuridine triphosphatase
(dUTPase) 792
Deoxyuridine triphosphate
(dUTP) 792
dFdCTP 1069
Diagnosis 566, 1139
Diets 95, 121, 1550
Differentials 725
Differential displays 1674
Differential splicing 63
1,25-Dihydroxyvitamin D3
550
Diphtheria toxins 1077
Diphtheria toxoid conjugate 956
Disease stages 196
Distant metastases 215
DNA damage 1209
DNA mismatch repairs 1643
DNA repairs 514, 1702
DNA strand breaks 69
DNA-dependent protein kinase
514
DNA-PK 1702
DNA-strand breaks 1740
Docetaxel 22
Drug combinations 146
Drug discovery paradigms 1401
Drug resistance 83, 921
Drug transport 375
DT-diaphorase 998
Dual topoisomerase inhibitors
1514
Dutch founder mutations 719
Dysplasia 1033
E6 gene 307
E-cadherin 209, 1168
Effect sizes 959
Elderly patients 573
EMF 692
-Emitters 1375
Endocrine factors 1234
Endocrine tumours 1003
Endometrial cancer 246, 1168,
1722, 1730
Endometrial hyperplasia 1722
Endothelin-1 360
Energy metabolism 1055
Environmental exposure 104
Enzyme-linked immunoassays
701
Ependymomas 407
Epidemiology 112, 387, 692,
952
Epigenetic deregulation 1583
Epithelial cells in bone marrow
35
Equivocal chest x-rays 1412
Equivocal liver echography
1412
Exit from G2/M 1084
Experimental therapeutics 817
Expression 498, 725
F 11782 1740
Familial isolated
hyperparathyroidism 1009
Familial predisposition 177
Fas 1715
Fas ligands 637
Fascin 870
FasL 1715
FDG 1607
18
F-FDG 281
FDG-PET 287
Ferns 914
Fern spores 69
-Fetoprotein isoforms 1330
Fibroblasts 346, 467, 650
Field carcinogenesis 1033
Flow cytometry 899
Fluctuation analysis 892
Fluorescence in situ
hybridization (FISH) 40, 1309
Fluorodeoxyglucose 863
Fluorouracil 1594
5-Fluorouracil 219, 594, 980
5-Fluorouracil, high-dose 1508
5-Fluorouracil sensitivity 1380
Folic acid deficiencies 1530
Folinic acid 219
Follicle stimulating hormone
(FSH) 1730
Follicular carcinomas 1494
Fruit 127
FWT1 177
FWT2 177
G1/S arrest 1380
G2/M override 346
Gastric cancer 387, 458, 986,
1026, 1394, 1681
Gemcitabine 447, 573, 707, 1069
Gene expression 209, 1047,
1154, 1494
Gene regulation 1192
Gene therapies 329, 1494
Gene-environment interaction
412
General breast cancer population
719
General Practice Research
Database 1554
Genes 246
Genetic polymorphisms 412,
1442
Genomic imbalances 1664
Genomic imprinting 1583
Genotypes 998
Glioblastoma 1281
Glioblastoma multiforme 588
Glioma 826
Glucose metabolism 1607
Glutathione 267, 375
Glycerol 1735
Glycosylation 1330
GnRH analogues 1730
Green fluorescent proteins 662
Growth 800
Growth factor 701
Growth inhibition 239, 1394
GS-X pump 375
Guided selections 252
H19 626
HaCaT-ras 1387
Haematogenous metastasis 324
Haemophilus influenzae type b
956
Haemophilus influenzae type b
polysaccharide vaccines 956
Head and neck cancer 30, 232,
421, 674, 775, 1696
Head and neck neoplasia 655
Health related quality of life 959
Helicobacter pylori 391
Hepatectomy 1096
Hepatoblastoma 1020
Hepatocellular carcinoma 50,
1096, 1330
Hepatocyte growth factor/scatter
factor 1147
HERG 1722
Herpes virus 329
Heterozygote detection 1650
High throughput screening 1047
High-dose-5-fluorouracil 1508
Histamine 826
Histological types 391
HMGI 1501
hMLH1 1643
hMSH2 1643
Hormonal therapy 443
Hormone receptor oestrogen
870
Hormone independency 782
Host-tumour interaction 519
4-HPR 333
HPV16 307
Human papillomavirus 1563
Human papillomavirus testing
561
17-Hydroxylase/C17,20
-lyase 74
17-Hydroxysteroid
dehydrogenase 550
Hypermethylation 1015
Hyperthermia 928
Hypoxia 354, 674, 899
I1307K polymorphism 153
IdUrd 899
IGF-II 626
IGFBP-rP2 756
Illudins 914
Image analysis 899
Immortality 156, 841
Immunity 1630, 1747
Immunodetection 204
Immunogenicity 519
Immunohistochemistry 215,
324, 498, 614, 620, 761,
1033, 1351, 1487, 1681, 1702
Immunomagnetic beads 1026
Immunosuppression 506
Immunotherapy 204, 1055,
1202
In situ carcinoma 729, 1472
In situ hybridization 1472
In vitro screens 1401
In vitro sensitivity 792
In vivo cancer chemotherapy
1514
In vivo expression 1467, 1589
Incidences 387, 391, 397, 952
Incidence rates 1554
Indirect calorimetry 1055
Infants 973
Infections 1557
Inflammatory bowel disease 89
Insulin-like growth factor-I 95
Insulin-like growth factor-II
1344
Integrins 156
Intercellular adhesion
molecule-1 847
Interferon 980
Interferon-a 219, 532, 583
Interleukin-1b 1442
Interleukin-2 826, 980, 1453
Interleukin-6 56
Interleukin-10 847
Interleukin-12 1536
Interleukin-15 526
Intestinal carcinoids 952
Intra-arterial bleomycin 1637
Intra-arterial chemotherapy
1637
Intramural metastasis 609
Intraperitoneal photodynamic
therapies 1542
Intravital microscopy 1216
Invading fronts 769
Invasion 1147
Ionizing radiation 514, 642,
1291
IQGAP1 1168
Irinotecan 146, 431
Japanese 1020
K-ras oncogenes 833
Karyotypes 1309
KDR/flk-1 1077
Keratinocytes 1387
Ki-67 (MIB-1) 1161
Ki-ras 473
Ku 1702
Lactadherin (BA46) 874
Lactose 1550
Laxatives 404
Leucocyte/lymphocyte
trafficking 1055
Leukaemia 956, 1571
Li-Fraumeni Syndrome 467,
1136
Linkages 1654
Localised breast cancer 577
Long-term quality of life 577
Longevity 1136
Longevity determination 841
Loss of heterozygosity 467, 729,
750, 1003
Loss of heterozygosity analysis
626
Low-dose hyper-radiosensitivity
514
LRP 921
Lung cancer 473, 632, 853, 858,
952
Luteinizing hormone 1730
Luteinizing hormone-releasing
hormone antagonists 906
Lymph nodes 184
Lymph node dissections 986
Lymph node metastases 609,
887, 1659
Lymphatic involvement 887
Lymphoblastic leukaemia, acute
756, 1617
Lymphocytes, rat 1530
Keyword index 1779
British Journal of Cancer (2000) 83(12), 1778-1781
(c) 2000 Cancer Research Campaign
Lymphoma cells 1375
MAGE-1 antigens 493
Magnetic fields 1571
Major histocompatibility
complex 1192
Male breast cancer 1234
Malignant cells 800
Malignant glioma 588
Malignant melanomas 1707
Malignant pericarditis 858
MALT-type lymphoma 454
Mammary carcinomas 1536
Mammographic parenchymal
patterns 121
Mammographies 1554
Manganese superoxide
dismutase 928
Markers 209
Mathematical models 98
Matrix metalloprotease
inhibitors 1536
Matrix metalloprotease-9 1536
Matrix metalloproteinase-2 215
MBD4 1646
MCF-7 270, 333
mdm2 1380
MDR-1 338
MDR1 Pgp 366
Meat eaters 95
Medullary thyroid carcinoma
715
Medullobastomas 407
MEK/ERK pathways 532
Melanomas 16, 184, 1216,
1351, 1447
Melanoma inhibitory activity
1216
Melanoma progression 847
Melphalan 1176
Membrane-type-1-matrix
metalloproteinase 215
Meningiomas 407
Menstrual cycle 1630
Mesothelioma 104, 853, 880,
1147
Meta-analysis 8, 692, 1688
Metallothionein 1E isoforms
319
Metastasectomy 569
Metastases 164, 354, 725, 973,
1747
Metastatic colorectal carcinoma
141
Metastatic gastric
adenocarcinoma 458
Methyl-donor deficiencies 1530
MHC-class II 1405
Micrometastasis 992
Microsatellite analysis 1707
Microsatellite instability 1646
Microvascular density 826
Microvessel density 196
Midkine 701
Milk fat globulin, human 874
Minimal residual cancer cells
1664
Misincorporated uracil 1530
Mismatch repairs 1291
Mitochondrial membrane
potential 1209
Mitoxantrone 91
MMAC1/PTEN/TEP1 1102
MMP 1147
Modulation 338
Molecular targets 1401
Molecular-based diagnosis 1494
MOLT-4 642
Monoclonal antibodies 498,
1542
Monoclonal antibody MA454
493
Mortality 841
Motility 870
mRNA 756
MRP1 366
Msh2 1291
MUC1 transgenic mice 1202
Mucins 874
Multidrug resistance 366, 892
Multidrug resistance protein 375
Multifocal involvement 454
Multiple endocrine neoplasia
type 1 1003, 1009
Multispecific organic anion
transporters 366
Multivariate analysis 35
Mutation analysis 1003, 1009
Mutations 737, 761
MYCN 40
MYCN 973
Mycobacterium vaccae 853
Myelodysplastic syndrome 91
Myeloid leukaemia, acute 91
L-NAME 1176
Nasopharyngeal carcinoma
1436, 1674
NAT2 412
Natural killers 1630, 1747
Necrosis 642
Necrosis factor-B 1367
Neoadjuvant chemotherapy
1479
Neovascularization 1154
Nested RT-PCR 184
Neuroblastic tumours 1501
Neuroblastoma 171, 973, 1124,
1295
Neuropeptide Y 171
Neuropeptide Y processing 171
Neuropeptide Y (3-36) 171
Neuropilin-1 1077
Neutropenia 594
Neutrophils 811
Nitric oxide 811
Nitric oxide synthase 880
nm23 1209
No55 743
Non-Hodgkin's lymphoma 1161
Non-seminomatous germ cell
tumours 1330
Non-small-cell lung cancer 447,
480, 573, 707, 1128
Non-small-cell lung cancer cell
lines 1069
Non-steroidal anti-
inflammatories 112
Normal cells 800
Norwegian founder mutations
1650
Nuclear localization 63
Nutrition 1550
Obesity 127
Oesophageal balloon cytology
1249
Oesophageal cancer 35, 127,
387, 689, 1249
Oesophageal squamous cell
carcinoma 1209
Oesophagus 1033
Oestradiol 550
Oestrogen 1231
Oestrogen receptors 311, 319
Oestrones 550
Oestrous cycle 1747
Oncogenes 1
Oncology 397
Oncolytic actovity 329
Oncoprotein 18 311
Oral cancer 1238
Oral hygiene 1238
Oral sex 1238
Organic anions 375
Oropharyngeal cancer 1594
Ovarian cancer 153, 404, 737,
1301, 1338, 1487
Ovarian cancer xenografts 921
Ovarian neoplasia 1550
Ovarian tumours 196, 906
Oxidized DNA bases 1530
Oxygen tension 354
p21 817
p21WAF1
1360
p21WAF/CIP1
50
p27 (Kip 1) 1161
p33ING1
1467
p53 50, 473, 761, 1033, 1084,
1136, 1287, 1338, 1360,
1467, 1735
p53 antibodies 1418
p53 genes 1039
p53 status 1380
Paclitaxel 83, 458, 1069, 1360,
1612, 1762
Pancreatic cancer 239, 329,
1442
Papillomavirus, human 1659
Pathological nodal metastasis
1479
Pathology 487, 1351
PCaP 1654
Pegylated liposomes 232, 684
Penetrance 1301
Penile cancer 1637
Performance indicators 421
Perfusion 1176
Pericardial effusion 858
Pericellular proteolysis 298
Peripheral stem cells 1405
Peritoneal lavage 1026
Peroxisome proliferator-
activated receptors- 1394
P-glycoprotein 892, 1762
Phaeochromocytomas 171
Phage displays 252
Phagocytosis 261
Pharmacokinetics 22, 74, 146,
232, 858, 935, 1599
Phase I trials 22, 146, 594
Phase II trials 438
Phenolphthalein 404
Phosphatidic acid 1599
Photochemotherapy 655
Photodynamic therapies 539,
1110, 1542
Photoimmunotherapy 1542
Photosensitizers 1542
Pimonidazole 674, 1523
Plasmin 298
Plasminogen activation system
1351
Pleura 880
Ploidy 40
Polarised cells 366, 375
Polyamine 594
Polylysine 1542
Polymerase chain reaction 1650,
1707
Polymorphism 307, 998, 1287
Pooled analysis 692
Population mixing 1557
Porphyrin biosynthesis 539
Positron emission tomography
863, 1607
Potassium channels 1722
Power lines 1571
PPAR- 1394
pRb 1161
Predictive assays 1702
Predictors 1447
Pregnancy 1231
Premenopausal node-negative
women 577
Preneoplasia 632
Prevalence 1301
Prioritization 1268
Procarbazine 588
Process 421
Prodrug 1589
Progesterone 870
Progesterone receptors 319,
1487
Progesterone receptors A 1487
Progesterone receptors B 1487
Prognosis 40, 164, 196, 209,
426, 473, 480, 487, 620, 986,
1033, 1323, 1338, 1351
Prognostic factors 431, 1442,
1617, 1696
Prognostic indicators 775
Prognostic markers 1688
Prohormone processing 171
Proliferation 30, 1318
Proliferation rates 566
Promoters 1015
ProNeuropeptide Y 171
Prostate cancer 74, 360, 443,
506, 519, 743, 992, 1102,
1431, 1654
Prostate-specific antigen nadir
1431
Proteasomes 526
Protein kinase C 782
Protein structures 1330
Protein truncation test 737
Protein turnover 526
Proteolysis-inducing factor 56
1780 Keyword index
British Journal of Cancer (2000) 83(12), 1778-1781 (c) 2000 Cancer Research Campaign
Keyword index 1781
British Journal of Cancer (2000) 83(12), 1778-1781
(c) 2000 Cancer Research Campaign
Psychosocial predictors 1447
Ptaquiloside 914
Pulmonary metastatic melanoma
569
Quality of life 447
Quantitative RT-PCR 626
Radiation 928
-Radiation 346
Radiation effects 1223
Radiation fibrosis 650
Radiation resistance 354
Radical retropubic prostatectomy
1431
Radioimmunoconjugates 1375
Radiosensitivity 1084, 1702,
1735
Radiotherapy 602, 650, 1281
RAF1 1084
Raltitrexed 146
Randomized trials 1128, 1243,
1594
Ras 156
Reactive oxygen species 941
Reassurance 1261
Recurrences 324, 986
Recurrent cancer 1696
5-Reductase 74
REG gene 188
Regeneration 188
Registries 952
Relapses 426, 1447
Renal cell carcinoma 583, 637,
980
Resistance 98, 338
Responses 98
Retinoblastoma genes 1039
Retinoic acid 1183, 1501
9-cis-Retinoic acid 239
13-cis-Retinoic acid 583, 1124
Retinoids 935
Retroperitoneal lymph node
dissections 1274
Reversal agents 366
Reverse transcriptase
polymerase chain reaction
992, 1323, 1487
Rhabdomyosarcoma 338
Ribozymes 833
Risk factors 689, 1231, 1550
Rituximab 1375
RNA in situ hybridization 1674
RT-PCR 319
RT-PCR-RFLP 998
S-1 141
S100A2 1472
S100A6 expression 769
Salvage 426
Sample sizes 959
Schedules 1612
Scotland 387
Screening 487, 1554
Second cancer 407
Second primary tumours 952
Sensitivity 928
SEREX 743
Serial analysis of gene
expression 725, 1494
Serine proteases 1351
Serological identification of
tumour antigens by
expression cloning 743
Serum 701
Serum CD26 1139
Serum-soluble interleukin-2
receptors 847
Sex hormones 95
Sexual practices 1238, 1563
Sialyl Lex
1681
Single-cell gel electrophoresis
(comet) assay 69
Sister-chromatid exchanges
1291
Skeletal muscle 526
Skin cancer 1243
SMAD4 1015
Small-cell lung cancer 8, 941,
1418
Smoking 412, 1234, 1563
Smoking cessation 689
SNH-3 1681
Socioeconomic status 387
Spectral karyotyping 1309
S-phase fraction 921
Sporadic endocrine tumours
1003
Squamous cell carcinomas 614,
655, 1367
SRL172 853
Stage IV 1128
Staging 454
Standards 421
Stathmin 311
Stealth liposomal doxorubicin
1281
Stomach neoplasia 391
Submucosal carcinoma of the
oseophagus 609
[Arg6
,D-Trp7,9
,Nme
Phe8
]-
Substance P (6-11) 941
Sunscreens 1243
Suppression subtractive
hybridization 1047, 1472
Surgical treatments 986, 1274
Surrogate measures 487
Survival 98, 397, 431, 614, 761,
952, 1447
Susceptibility 1643
Systematic reviews 561
Tamoxifen 16
Tamoxifen resistance 782
Taxanes 438, 1762
T-cell activation 1453
Tegafur 141
Telomerases 841
Telomeres 841
Telomeric repeat amplification
protocol assay 1026
Temozolomide 588
Teratoma 426
Tertile 121
Testicular cancer 863
Testicular germ cell tumours
729
Testicular neoplasia 1274
Thymidine phosphorylase 219
Thymidylate synthase 792
Thymidylate synthase
suppression 1508
Time trends 952
TIMP 1147
TIMP-1 1536
Tirapazamine 1589
Tissue factors 164
Tissue inhibitors of
metalloproteinase-2 215
Tissues 498
Topoisomerase II 1589
Topoisomerase IIIa 498
Toxicity 431
TP53 467
tPA 1351
Transcription factors 1192, 1367
Transfection 1039
Transforming growth factor-
480, 650
Transforming growth factor-
expression 519
Transgenic mice 1
Transitional cells 1612
Treatments 98, 602, 1268
Tumorigenesis 63, 156, 1154
Tumours 261
Tumour-associated antigens 204
Tumour blood flow 225
Tumour cell invasion 1216
Tumour cell involvement 1323
Tumour conditions 662
Tumour growth 1096
Tumour hypoxia 1523
Tumour imaging 1607
Tumour incidences 519
Tumour-infiltrating lymphocytes
637, 1715
Tumour markers 701, 1412,
1418, 1696, 1722
Tumour microcirculation 1055
Tumour necrosis factor 1176
Tumour necrosis factor- 480,
1367
Tumour oxygenation 225
Tumour perfusion 225, 899
Tumour progression 1707
Tumour recurrence 1139
Tumour suppressor genes 1,
473, 750, 1467, 1583
Tumour targeting 232, 684
Tumour vasculature 225
Turkish population 737
Turkoman Plain 1249
Twins 1231
Type IV collagenases 1387
Tyrosinase 184
Ubiquitin 526
Ulcerative colitis 89
Ultraviolet radiation 1243, 1467
Unfit patients 573
Urokinase plasminogen
activators 298
UVc 346
Vaccinations 956
Vascular density 674
Vascular endothelial growth
factor 63, 196, 620, 775,
1077
Vascular endothelial growth
factor-C 887
Vascularity 684
Vasculature 811
Vegans 95
Vessel invasion 609
Vinblastine 892, 1612
Vinorelbine 573, 707
Vitamin D analogues 239
Vulval cancer 1287
Vulval intraepithelial neoplasia
1287
Warts 1238
Western blot 311, 775
Wilm's tumour 177, 602
Xenogeneic cells 1453
Xenograft tumours 232
Xenografts 684
Yoshida sarcoma 1055
Young adults 397
ZD2767 267
ZD9331 792

				

